# Dog ownership and adults’ objectively-assessed sedentary behaviour and physical activity

**DOI:** 10.1038/s41598-020-74365-6

**Published:** 2020-10-15

**Authors:** Mohammad Javad Koohsari, Ai Shibata, Kaori Ishii, Sayaka Kurosawa, Akitomo Yasunaga, Tomoya Hanibuchi, Tomoki Nakaya, Gavin R. McCormack, Koichiro Oka

**Affiliations:** 1 Faculty of Sport Sciences, Waseda University, 2-579-15 Mikajima, Tokorozawa, Saitama 359-1192 Japan; 2 Behavioural Epidemiology Laboratory, Baker Heart and Diabetes Institute, Melbourne, Australia; 3 Melbourne School of Population and Global Health, The University of Melbourne, Melbourne, Australia; 4 Faculty of Health and Sport Sciences, University of Tsukuba, Tsukuba, Japan; 5 Graduate School of Sport Sciences, Waseda University, Tokorozawa, Japan; 6 Faculty of Liberal Arts and Sciences, Bunka Gakuen University, Tokyo, Japan; 7 Graduate School of Environmental Studies, Tohoku University, Sendai, Japan; 8 Department of Community Health Sciences, Cumming School of Medicine, University of Calgary, Calgary, Canada; 9 Faculty of Kinesiology, University of Calgary, Calgary, Canada; 10 School of Architecture, Planning and Landscape, University of Calgary, Calgary, Canada

**Keywords:** Epidemiology, Risk factors

## Abstract

Evidence suggests a positive effect of dog ownership on physical activity. However, most previous studies used self-reported physical activity measures. Additionally, it is unknown whether owning a dog is associated with adults’ sedentary behaviour, an emerging health risk factor. In this study, physical activity and sedentary behaviour were objectively collected between 2013 and 2015 from 693 residents (aged 40–64 years) living in Japan using accelerometer devices. Multivariable linear regression models were used, adjusted for several covariates. The means of total sedentary time and the number of long (≥ 30 min) sedentary bouts were 26.29 min/day (95% CI − 47.85, − 4.72) and 0.41 times/day (95% CI − 0.72, − 0.10) lower for those who owned a dog compared to those not owning a dog, respectively. Compared with non-owners, dog-owners had significantly higher means of the number of sedentary breaks (95% CI 0.14, 1.22), and light-intensity physical activity (95% CI 1.31, 37.51). No significant differences in duration of long (≥ 30 min) sedentary bouts, moderate, vigorous, and moderate-to-vigorous-intensity physical activity were observed between dog-owners and non-owners. A novel finding of this study is that owning a dog was associated with several types of adults’ sedentary behaviours but not medium-to-high-intensity physical activities. These findings provide new insights for dog-based behavioural health interventions on the benefits of dog ownership for reducing sedentary behaviour.

## Introduction

Pet ownership, particularly dog ownership, is associated with physical and mental health benefits^1,2^. For example, a nationwide cohort study conducted in Sweden found that dog ownership was associated with a lower risk of cardiovascular disease and mortality^3^. Another national study conducted in the USA found that pet ownership (either cat or dog ownership) was associated with lower odds of systemic hypertension^4^. Dog walking is one of the possible mechanisms through which dog ownership may affect people’s physical activity and health^5,6^. The health benefits of physical activity have been well-documented^7^, yet most adults are insufficiently active^8^. In the USA, only 22.9% of adults met national guidelines for aerobic and muscle-strengthening activities^9^. In Japan, population-level physical activity has continuously decreased during the last few decades^10^, and about 38% of Japanese were insufficiently active in 2016, according to the World Health Organisation report^11^. Alongside individual-based factors, socio-ecological models emphasise the role of social and urban design factors in supporting physical activity^12^. Given the prevalence of dog ownership in households in Japan (17%)^13^ and other countries such as Canada (35%)^14^, USA (48%)^15^ and Australia (38%)^16^, physical activity promotion strategies that target dog-owners could have a significant impact on population health.

There is growing evidence over the positive effects of dog ownership on people’s physical activity^17–19^. For instance, a study conducted in the UK found that dog-owners were more likely to undertake more recreational walking and have higher odds of meeting physical activity guidelines, compared with non-owners^20^. Another study conducted in Finland found that dog ownership was associated with higher recreational physical activity in late adulthood^21^. However, this area of research has been limited in several important ways. Most previous studies examining dog ownership and active behaviour used self-reported physical activity measures. A systematic review identified only four studies (out of 29 studies) that used objective measures of physical activity to examine physical activity among dog-owners and non-owners^17^. While self-reported measures of physical activity provide important contextual information, they are limited in some respects. First, self-reported measures of physical activity are subject to recall bias since most people have difficulty in recalling their accurate levels of activities^22^. Second, because physical activity is considered a socially desirable activity, people tend to overreport their physical activity^23^. Finally, self-reported measures of physical activity are unable to accurately capture absolute or total levels of physical activities^24^. To address these issues, there is a need to include objective measures of physical activity, such as accelerometers, in studies comparing time spent in total and intensity-specific physical activity by dog ownership status. Notably, some previous studies used objective physical activity measures in relation to dog ownership using accelerometer devices^25–28^. For example, a study conducted in the USA found a positive association between dog ownership and adolescents’ physical activity estimated by accelerometer devices^25^. Another study using accelerometer devices conducted in the UK found that dog walkers were more active than non-owners among a sample of older adults^26^. Nevertheless, these studies are mainly conducted in Western countries, and there is a lack of studies in Asia, where pet ownership is rising^29^. Cultural differences in dog ownership and view of dogs in different geographical locations may impact physical activity decision^30,31^. Additionally, few studies exist examining whether owning a dog may be associated with objectively-measured sedentary behaviour^25,32,33^. Sedentary behaviour is an emerging health risk factor independent of physical activity^34–37^ For example, a recent systematic review of prospective studies found that sitting time was associated with an increased risk of cardiovascular disease and diabetes, regardless of physical activity levels^36^. Another systematic review found that total sitting and TV viewing time were associated with higher risk for several major chronic diseases, independent of physical activity^37^. Dog ownership may affect health by reducing people’s sedentary behaviour^32,38^. In most of the previous studies, however, self-reported sedentary behaviour was included^39^. For instance, a study conducted in Australia found that dog ownership was not significantly associated with self-reported screen time among children^40^. Further research is needed to explore whether owning a dog may influence adults’ objectively-assessed sedentary behaviour or not.

Therefore, our study aimed to investigate the extent to which dog ownership was associated with objectively-assessed sedentary behaviour and physical activity among adults (40–64 years) living in two Japanese urban areas.

## Results

Complete data from 693 participants were included in this analysis. Our sample included 119 (17.2%) dog-owners. The mean age was 52.2 years, and about 61% were female, about two-thirds (64.1%) had completed tertiary education, about 82% were employed, and approximately 45% had an annual gross household income lower than ¥5,000,000 (≈ USD 50,000) (Table 1). The mean accelerometer wearing time was about 15.4 (SD = 1.5) hours per day, with an average of approximately 7.0 (SD = 0.9) days. Socio-demographic characteristics and accelerometer wearing time/days did not significantly differ between dog-owners and non-owners. Table 2 shows the objectively-assessed sedentary behaviour and physical activity of dog-owners and non-owners. Among dog-owners, total sedentary time, duration, and the number of long (≥ 30 min) sedentary bouts were significantly (p < 0.05) lower compared with non-owners. Sedentary breaks and duration of light-intensity physical activity (min/day) were significantly higher among dog-owners than non-owners. There was no statistically significant difference in moderate, vigorous, and moderate-to-vigorous physical activity between these two groups.

**Table 1:** Characteristics of study participants by dog ownership status (n = 693).

Variable	Mean (S.D.) or N (%)		
	Total	Dog-owners (n = 119)	Non-owners (n = 574)
Age (years)	52.2 (7.1)	52.5 (6.9)	52.1 (7.1)
**Gender**			
Female	424 (61.2)	72 (60.5)	352 (61.3)
Male	269 (38.8)	47 (39.5)	222 (38.7)
**Highest education**			
Tertiary	444 (64.1)	67 (56.3)	377 (65.7)
Below tertiary	249 (35.9)	52 (43.7)	197 (34.3)
**Working status**			
Employed	570 (82.3)	103 (86.6)	467 (81.4)
Unemployed	123 (17.7)	16 (13.4)	107 (18.6)
**Gross annual household income**			
< ¥5,000,000	315 (45.5)	55 (46.2)	260 (45.3)
≥ ¥5,000,000	378 (54.5)	64 (53.8)	314 (54.7)
**Municipality**			
Koto ward	336 (48.5)	39 (32.8)	297 (51.7)
Matsuyama city	357 (51.5)	80 (67.2)	277 (48.3)
Accelerometer wearing days	7.0 (0.9)	7.1 (0.9)	7.0 (0.9)
Accelerometer wearing time (min/day)	921.2 (90.8)	926.0 (93.7)	920.2 (90.3)

No statistically significant difference between dog-owners and non-dog owners based on independent t-tests and Pearson’s chi-square test, except for municipality.

**Table 2:** Sedentary behaviour and physical activity of dog-owners and non-owners (n = 693).

	Mean (S.D.)	
	Dog-owners (n = 119)	Non-owners (n = 574)
Total sedentary time (min/day)*	473.1 (129.9)	506.3 (117.6)
Duration of long (≥ 30 min) sedentary bouts (min)*	155.7 (99.0)	175.9 (94.0)
Number of long (≥ 30 min) sedentary bouts (times/day)*	2.9 (1.6)	3.4 (1.6)
Sedentary breaks (times/sedentary hour)*	10.1 (3.2)	9.2 (2.7)
Light-intensity physical activity (min/day)*	376.9 (115.6)	344.7 (109.1)
Moderate-intensity physical activity (min/day)	74.0 (40.1)	67.3 (37.4)
Vigorous-intensity physical activity (min/day)	1.9 (8.8)	1.9 (5.6)
Moderate-to-vigorous-intensity physical activity (min/day)	75.9 (41.7)	69.2 (38.7)

Sedentary behaviour, light-intensity physical activity, moderate-intensity physical activity, vigorous-intensity physical activity, and moderate-to-vigorous physical activity were defined with an estimated accelerometer intensity of ≤ 1.5 METs, 1.6 to 2.9 METs, 3.0–5.9 METs, ≥ 6.0 METs, and ≥ 3.0 METs, respectively.

*p-values based on independent t-tests.

Adjusting for covariates, the means of total sedentary time and the number of long (≥ 30 min) sedentary bouts were 26.29 min/day (95% CI − 47.85, − 4.72) and 0.41 times/day (95% CI − 0.72, − 0.10) lower for those who owned a dog compared to those not owning a dog, respectively. Compared with non-owners, dog-owners had significantly higher means of the number of sedentary breaks (95% CI 0.14, 1.22), and light-intensity physical activity (95% CI 1.31, 37.51). No significant differences in the duration of long (≥ 30 min) sedentary bouts, moderate, vigorous, and moderate-to-vigorous-intensity physical activity were observed between dog-owners and non-owners (Table 3).

**Table 3:** Associations of dog ownership with objectively-assessed sedentary behaviour and physical activity (n = 693).

	Unadjusted *B* (95% CI)	Adjusted *B* (95% CI)
Total sedentary time (min/day)	− 33.14 (− 56.83, − 9.44)*	− 26.29 (− 47.85, − 4.72)*
Duration of long (≥ 30 min) sedentary bouts (min)	− 20.21 (− 38.98, − 1.45)*	− 16.19 (− 34.68, 2.30)
Number of long (≥ 30 min) sedentary bout (times/day)	− 0.48 (− 0.80, − 0.17)*	− 0.41 (− 0.72, − 0.10)*
Number of sedentary breaks (times/sedentary hour)	0.88 (0.33, 1.43)*	0.68 (0.14, 1.22)*
Light-intensity physical activity (min/day)	32.21 (10.42, 54.01)*	19.41 (1.31, 37.51)*
Moderate-intensity physical activity (min/day)	6.67 (− 0.82, 14.16)	6.54 (− 0.92, 14.01)
Vigorous-intensity physical activity (min/day)	0.02 (− 1.21, 1.26)	0.33 (− 0.90, 1.57)
Moderate-to-vigorous-intensity physical activity (min/day)	6.69 (− 1.06, 14.44)	6.88 (− 0.85, 14.61)

All models adjusted for age, gender, highest education, working status, gross annual household income, municipality, and accelerometer wearing time. Reference group: non-dog owners.

*B* regression unstandardised coefficient, *CI* confidence interval.

*p < 0.05.

## Discussion

We examined the associations between dog ownership with objectively-assessed sedentary behaviour and physical activity among a sample of middle-aged adults in Japan. We found that dog ownership was negatively associated with total sedentary time and number of long sedentary bouts, and positively associated with number of sedentary breaks and light-intensity physical activity. Our findings are consistent with a previous study, which used self-reported measures of sedentary behaviour^39^. Oka and Shibata^39^ found that dog ownership was associated with less daily sedentary behaviour in adults. Some previous studies conducted among older adults also support our findings in part. For instance, owning a dog was associated with a lower likelihood of sitting over 8 h per day among older women^38^. Another study found that dog-owners had fewer sitting events compared with non-owners. However, they found no significant associations between owning a dog and total sitting time or duration of prolonged sitting events^32^. Our study supports these findings by showing that dog ownership was not only negatively associated with objectively-assessed total sedentary time but with prolonged bouts of sitting. Evidence suggests that both total and prolonged bouts of sitting have adverse health effects^41,42^. Dog ownership may create opportunities for their owners to break their prolonged sedentary behaviour and be engaged in light-intensity physical activities for daily caring for their dogs.

Our findings are in contrast with some previous studies conducted among other age groups. For example, a previous study found that owning a dog was not significantly associated with accelerometer-derived sedentary time in a sample of adolescents^25^. Another study found that dog ownership was significantly associated with fewer sitting events in older adults, but not with either the total sitting time or the number or duration of prolonged sedentary events^32^. These findings highlight that dog ownership may have different effects on sedentary behaviours in different age groups. Further research is needed to track how dog ownership may influence sedentary behaviours patterns across the life course.

We found no significant difference in moderate, vigorous, and moderate-to-vigorous-intensity physical activities between dog-owners and non-owners. These findings are in contrast with some previous studies which used self-reported physical activity measures or were conducted in other age groups^20,21,39,43^. A study conducted in Melbourne, Australia found that owning a dog was associated with higher moderate-to-vigorous-intensity physical activity among children^43^. Another study found that dog-owners were more likely to achieve sufficient levels of physical activity and walking compared with non-owners^44^. Moreover, a study found that dog ownership was associated with higher life course and leisure-time physical activity in late adulthood^21^. There may be several reasons for these inconsistent findings between studies. While much of the physical activity benefits of owning a dog may confer from dog walking, not all dog-owners walk their dogs^5^. The proportion of dog-owners who walked their dogs widely varied between previous studies. For instance, a study conducted in Canada found about 16% of dog-owners did not walk their dogs^45^. Another study conducted in the USA reported about 25% of their participants as dog-owners non walkers^46^. A recent study of Japanese adults showed that about 30% of dog-owners did not walk their dogs in a usual week^47^. Additionally, there might be socio-cultural or urban design factors that act to discourage dog-owners from walking their dogs in the context of our Japanese adults. Since Japan has one of the most prolonged working hours in the world^48^, dog-owners may have limited time to devote to walking and playing outside with their dogs. Unique urban design attributes of Japanese urban environments such as narrow sidewalks, small housing, and lack of dog-friendly parks may also deter dog-owners from undertaking dog-related medium-to-high intensity physical activities. Furthermore, and relevant to the compact built environment, small dog breeds are popular among dog-owners in Japan^49^. According to the Japan Pet Food Association’s survey, more than half of Japanese dog-owners have a small-sized dog^50^. Such breeds may require less physical activities compared with their larger breeds^51^. Further studies are needed to identify correlates of higher intensity physical activities among dog-owners in this context.

Our study has some limitations. As a cross-sectional study, it cannot provide strong causal evidence. Dog characteristics (such as age, sizes, breeds) and owner-dog attachment or bond were not considered in this study. Objective measures of sedentary behaviour and physical activity do not provide contextual information on the specific types of these activities. Therefore, the extent to which dog-owners physical activity included dog walking was not captured. A key strength of this study is the use of objectively-assessed sedentary behaviour and physical activity. The examination of physical activity at different intensities is another strength of this study.

## Conclusions

This study adds to the existing literature on dog ownership and active and sedentary behaviours by using objectively-assessed behaviours. Our findings can contribute to a better understanding of how dog ownership may impact adults’ active and sedentary behaviours. A novel result of this study is that owning a dog was associated with several types of adults’ sedentary behaviours but not medium-to-high-intensity physical activities. These findings provide several new insights for dog-based behavioural health interventions: first, they highlight the importance of dog ownership for reducing adults’ sedentary behaviour, as an emerging health risk. Dog ownership status appears to be associated with frequency of bouts and time spent sedentary. Notably, a previous review reported the importance of various strategies to encourage dog walking in order to improve health^18^. However, our findings suggest that lower levels of sedentary behaviour, in addition to increased physical activity accumulated via dog walking, might be another pathway by which dog owners accumulate health benefits. Second, our findings indicate that other factors, as well as dog ownership, are necessary to affect dog owners’ medium-to-high-intensity physical activities. Finally, dog ownership status is an important correlate of physical activity and sedentary behaviour in Japanese middle-aged adults and therefore should be considered in the design and planning of health interventions and in studies investigating the determinants of physical activity and sedentary behaviour in this target population.

## Methods

### Study and sample design

Our analysis included cross-sectional data from an epidemiological study undertaken to examine social and urban design correlates of sedentary behaviour and physical activity among Japanese adults aged 40–64 years. Detailed methods of study design and recruitment have been documented elsewhere^52^. Briefly, data were obtained between July to December 2013 and April 2014 to February 2015 from residents living in two Japanese urban localities, Koto Ward and Matsuyama City. To recruit participants, an invitation letter was sent to 6,000 adult residents (aged 40–64 years), randomly selected from the government registry of residential addresses. About two weeks after the initial mailing, a reminder letter was sent to non-respondents. A self-administered questionnaire and an accelerometer (with a log diary) were posted to the 866 individuals (response rate = 14.4%), who agreed to participate in this study. Of these, 779 completed the questionnaire and returned the accelerometers. A book voucher (¥1000 equivalent to about USD$10) was given to these participants. The study was approved by the Research Ethics Committee, Waseda University, Japan (2012-269) and the methods were carried out in accordance with these guidelines. Informed consent was obtained from each participant.

### Measures

#### Sedentary behaviour and physical activity

Sedentary behaviour and physical activity were objectively-assessed using a validated tri-axial accelerometer (Active style Pro model HJA-350IT; Omron Healthcare, Kyoto, Japan)^53,54^. Participants were asked to wear the accelerometer on their waist for at least seven days while they are awake, except during water-based activities (e.g., bathing, showering, swimming). Participants who wore the accelerometer for at least four days (including one non-working day), with at least 10 h/day of wear time per day, were eligible for this study^55^. The data were collected in 1-min epochs and expressed as metabolic equivalents (METs). Sedentary behaviour, light-intensity physical activity, moderate-intensity physical activity, vigorous-intensity physical activity, and moderate-to-vigorous physical activity were defined with an estimated accelerometer intensity of ≤ 1.5 METs, 1.6 to 2.9 METs, 3.0–5.9 METs, ≥ 6.0 METs, and ≥ 3.0 METs, respectively^56,57^. Four sedentary behaviour outcomes, including total sedentary time, duration and number of long (≥ 30 min) sedentary bouts and breaks per sedentary hour, were calculated. The total sedentary time per day was obtained by summing the time spent engaged in any sedentary behaviour^57^. Sedentary bouts were defined as periods of uninterrupted sedentary time and a sedentary bout referred to at least 30 consecutive minutes of sedentary time^57,58^. A sedentary break was defined as a non-sedentary bout between two sedentary bouts^57^. Physical activity outcomes were averages of light, moderate, vigorous, and moderate-to-vigorous-intensity physical activity per day.

#### Dog ownership

Participants were asked whether they currently own a pet in their household or not. Those who owned a pet reported the type of pet, including dog, cat, and others (e.g., birds, fish, and reptile). We compared those with (owners) and without (non-owners) a dog at home only.

#### Covariates

Participants reported the following socio-demographic characteristics: age, gender (female versus male), highest educational attainment (tertiary versus below tertiary), working status (employed versus unemployed), and annual gross household income (< ¥5,000,000 versus ≥ ¥5,000,000). The municipality (Koto Ward versus Matsuyama City) and daily accelerometer wearing time were also included as covariates.

### Statistical analysis

Descriptive statistics including frequencies and measures of central tendency and variation (i.e., means, standard deviations) were estimated for socio-demographic, dog ownership, sedentary behaviour, and physical activity variables. Independent t-tests and Pearson’s chi-square test were used to compare these variables between dog-owners and non-owners. Multivariable linear regression models were used to estimate associations of dog ownership with objectively-assessed sedentary behaviour and physical activity. All models adjusted for the covariates. Analyses were conducted using Stata 15.0 (Stata Corp, College Station, Texas), and the level of significance was set at p < 0.05.
